# Global Prevalence and Risk of Local Recurrence Following Cryosurgery of Giant Cell Tumour of Bone: A Meta-Analysis

**DOI:** 10.3390/cancers14143338

**Published:** 2022-07-08

**Authors:** Shyful Nizam Sumari, Nor Azman Mat Zin, Wan Faisham Wan Ismail, Md Asiful Islam

**Affiliations:** 1 Department of Orthopaedics, School of Medical Sciences, Universiti Sains Malaysia, Kubang Kerian 16150, Kelantan, Malaysia; shyfulnizamsumari@gmail.com (S.N.S.); faisham@usm.my (W.F.W.I.); 2 Department of Orthopaedics and Traumatology, Hospital Sungai Buloh, Sungai Buloh 47000, Selangor, Malaysia; 3 Department of Haematology, School of Medical Sciences, Universiti Sains Malaysia, Kubang Kerian 16150, Kelantan, Malaysia; 4 Institute of Metabolism and Systems Research, University of Birmingham, Birmingham B15 2TT, UK

**Keywords:** giant cell tumour, local recurrence, cryosurgery, prevalence, risk

## Abstract

**Simple Summary:**

Giant cell tumours are benign but locally aggressive and can potentially metastasise to the lungs. Reducing the risk of local recurrence while maintaining limb function and minimising adverse consequences is the best therapeutic strategy in treating giant cell tumours. Based on our observation through this meta-analysis, cryosurgery is one of the viable treatment options that can provide good oncologic and functional outcomes with minimal complication rates.

**Abstract:**

The challenge in the surgical treatment of giant cell tumours of bone is the relatively high recurrence rate after curettage alone. The use of a local adjuvant following curettage, on the other hand, has lowered the rate of recurrence. This systematic review and meta-analysis aimed to investigate the prevalence and risk of local recurrence of giant cell tumours of the bone after cryosurgery and the subsequent complications. Web of Science, Scopus, ScienceDirect, PubMed, and Google Scholar were searched to identify articles published until 13 October 2021. A random-effects model was used to examine the pooled prevalence and risk ratio (RR) of local recurrence in patients with giant cell tumours after cryosurgery with 95% confidence intervals (CIs). This study was registered with PROSPERO (CRD42020211620). A total of 1376 articles were identified, of which 38 studies (n = 1373, 46.2% male) were included in the meta-analysis. Following cryosurgery, the pooled prevalence of local recurrence in giant cell tumours was estimated as 13.5% [95% CI: 9.3–17.8, *I*^2^ = 63%], where European subjects exhibited the highest prevalence (24.2%). Compared to other local adjuvants. The RR of local recurrence following cryosurgery was 0.85 (95% CI: 0.63–1.17, *I*^2^ = 15%), which was not statistically significant compared to other local adjuvants. We found 3.9% fracture, 4.0% infection, 2.1% nerve injury, and 1.5% skin necrosis as the common complications. Based on the sensitivity analyses, this study is robust and reliable. This meta-analysis estimated a low prevalence of local recurrence of giant cell tumours with low complications following cryosurgery. Thus, it can be one of the adjuvant options for treating giant cell tumours.

## 1. Introduction

Bone giant cell tumours account for approximately 5% of all primary bone lesions and 20% of benign bone tumours. It is a benign bone lesion that is aggressive locally and seldom metastasises [1,2]. Reactive multinuclear osteoclast-like giant cells expressing receptor activator of nuclear factor k-B (RANK) and neoplastic mononuclear stromal cells expressing RANK-ligand (RANKL) generate giant cell tumours, which lead to osteoclast formation and bone resorption [2].

The symptoms of giant cell tumours are non-specific, such as pain, reduction in the affected joint’s range of motion, and swelling. The pathological fracture is usually associated with acute onset of pain, and the incidence of pathological fracture is approximately 10–22% [3,4,5,6,7]. The symptoms of giant cell tumours of the sacrum are usually subtle. The tumours remain silent until their slow progress reaches a critical size, causing the symptoms such as lower back pain, radiating pain to the legs, change in sexual dysfunction, and bladder and bowel patterns [8]. Most research found that female patients were more likely than male patients to develop giant cell tumours, with ratios ranging from 1:1.1 to 1:1.5. The meta-epiphyseal region of the long bone accounts for 75% to 90% of giant cell tumours, with 84% to 99% of lesions spreading to the subarticular region within 1 cm. The common location of the lesion is the distal femur, followed by the proximal tibia [9].

Based on the radiological appearance, giant cell tumours are divided into three grades: (i) A grade 1 lesion (latent) has a well-defined boundary and no cortical disruption; (ii) an active grade 2 lesion has a thinning cortex, expansile, and well-defined border; and (iii) an aggressive grade 3 lesion has cortical destruction and unclear boundaries [10]. Enneking et al. [11] suggested another classification based on histo-radiological features: (i) stage 1 (latent) refers to asymptomatic patients with a well-defined margin on radiograph, histologically benign lesion; (ii) stage 2 (active) refers to symptomatic patients with an expansile cortex with no cortical disruption, histologically benign lesion; (iii) stage 3 (aggressive) refers to symptomatic patients with a rapidly growing lesion, cortical disruption with soft tissue mass, may metastasise, histologically benign lesion; and (iv) a sarcomatous lesion contiguous with a benign giant cell tumour is classified as stage 4 (malignant). According to the Campanacci grading system, grade 1 and grade 2 lesions should be treated with intralesional curettage, and grade 3 lesions should be treated with en bloc resection and reconstruction if required [12]. However, whether these classifications reliably reflect the aggressiveness of GCT or provide prognostic value in terms of local recurrence rates and functional outcomes is questionable.

Surgical treatment options for giant cell tumours of the bone ranged from curettage to wide excision, with variable outcomes [13]. A high local recurrence rate is the most challenging complication following surgical curettage alone. Surgical resection has the lowest recurrence rates but is associated with functional impairment [14]. Curettage with local adjuvants is, therefore, the recommended therapeutic approach since it has a better functional result, reduced morbidity, and a low incidence of local recurrence [15]. Phenol, hydrogen peroxide, ethanol, liquid nitrogen, polymethylmethacrylate (PMMA), and argon beam are examples of local adjuvants used to minimise local recurrence. Furthermore, in recent years, the neoadjuvant and adjuvant roles of denosumab in GCT have demonstrated interesting results by changing the therapeutic paradigm of GCT.

To our knowledge, there has never been a systematic review and meta-analysis of local recurrence in giant cell tumours following cryosurgery. Thus, the objective of this study was to estimate the prevalence and risk of local recurrence of giant cell tumours of the bone treated with cryosurgery compared to other therapies and to assess the complications of cryosurgery in treating giant cell tumours.

## 2. Methods

This systematic review and meta-analysis were carried out using the Preferred Reporting Items for Systematic Review and Meta-Analysis (PRISMA) guidelines and recommendations to estimate the prevalence and risk of local recurrence of giant cell tumours of bone following cryosurgery [16]. With the registration number CRD42020211620, the research protocol was registered to PROSPERO (an international database of prospectively registered systematic reviews) at the University of York, UK.

### 2.1. Data Sources and Searches

The bibliographic databases Web of Science, Scopus, Science Direct, PubMed, and Google Scholar were combed for papers published until 13 October 2020. The following key terms were used to construct the search strategies: cryosurgery, cryosurgeries, cryoablation, cryoablations, cryotherapy, cryotherapies, adjuvant, liquid nitrogen, giant cell tumour, giant cell tumours, giant cell tumour and giant cell tumours (Table S1). 

### 2.2. Eligibility Criteria and Study Selection 

Patients of any sex, age, or race from observational studies documenting the prevalence of local recurrence of giant cell tumours of the long, small or flat bone following cryosurgery with no language barrier were deemed eligible. Review articles, clinical trials, editorials, comments, case reports, and studies on non-human subjects were excluded. EndNote X8 software (Clarivate Analytics, Philadelphia, PA, USA) was used to eliminate duplicate studies from various databases. In addition, references in the primary articles were checked to determine whether there was any additional relevant study. Two authors (S.N.S. and M.A.I.) screened an article’s title and abstract and selected it based on the eligibility criteria. Disagreements concerning inclusion were discussed, and a consensus was reached by discussing with the third author (N.A.M.Z.). 

### 2.3. Data Extraction and Quality Assessment 

Two authors (S.N.S. and M.A.I.) extracted the following data and information from each eligible article into a prepared Excel spreadsheet separately: first author’s last name; study duration; location of the participants; mean age; the total number of giant cell tumours; mean follow up time and Campanacci grading. The random-effects model was used to analyse the pooled prevalence and 95% confidence intervals (CIs) of local recurrence in patients with giant cell tumours following cryosurgery.

Two authors (S.N.S. and M.A.I.) independently assessed the quality of the included studies using the Joanna Briggs Institute (JBI) critical appraisal tool [17]. The studies were categorised as high quality (low risk of bias) if the overall score was equal to or more than 70%, moderate quality (moderate risk of bias) if the overall score was 50–69%, and low quality (high risk of bias) if the total score was less than 50% [18,19]. In addition, a funnel plot was constructed if there was a minimum of ten studies estimating the prevalence against the standard error to analyse publication bias via visualising asymmetry. Additionally, Egger’s test was used.

### 2.4. Data Syntheses and Analysis

*I^2^* statistics and Cochran’s Q-test were used to assess study heterogeneity. The *I*^2^ value of >75% and a significance level of 0.05 indicated significant heterogeneity [20]. Additionally, a Galbraith plot was constructed to identify the sources of heterogeneity and the outlier studies. Subgroup analyses were done based on the patients’ mean age range and the participants’ location. Sensitivity analysis was conducted, excluding the outlier studies. RevMan (version 5.4) and metaprop codes in meta (version 4.19-0) and metafor (version 3.0-2) packages of R (version 3.6.3) in RStudio (RStudio, Inc., Boston, MA, USA) (version 1.4.1106) software were used to analyse and generate plots [21].

## 3. Results

### 3.1. Study Selection

**Figure 1:**
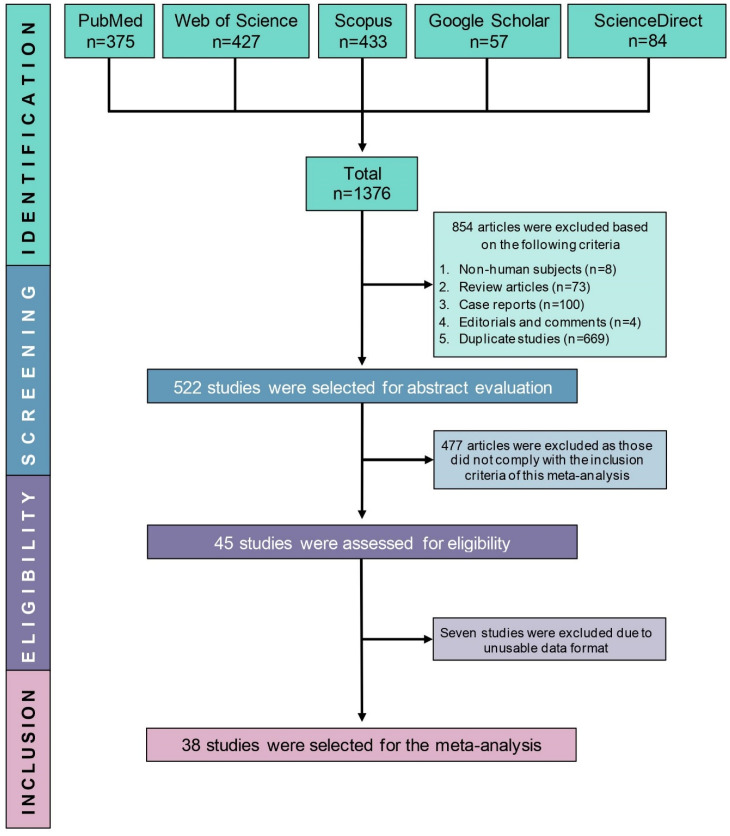
PRISMA flow diagram of study selection.

We initially found 1376 articles from five bibliographic databases based on the search strategy. Eight hundred fifty-four articles were excluded in the identification phase (duplicate studies, n = 669; case reports, n = 100; review articles, n = 73; non-human studies, n = 8; editorial and comment, n = 4), and the remaining 522 articles were further examined. A total of 477 articles were excluded as those did not comply with the study objective, and seven were further excluded due to unusable data format. As a result, 38 articles were finally included in this systematic review and meta-analysis, and full texts of all 38 articles were obtained (Figure 1).

### 3.2. Study Characteristics

Our literature search yielded 38 observational studies [4,5,6,7,22,23,24,25,26,27,28,29,30,31,32,33,34,35,36,37,38,39,40,41,42,43,44,45,46,47,48,49,50,51,52,53,54,55] published between 1949 and 2016, which examined the outcome of cryosurgery in patients with giant cell tumours of the bone. Table 1 shows the detailed features of the included articles. In total, 1373 individuals with giant cell tumours (92.2% benign GCT and 7.8% malignant GCT) where 672 patients were treated with cryosurgery were studied in this meta-analysis (46.2% male). The mean age of the giant cell tumour patients was 32.7 years ranging from 20.0 to 42.4 years. Included articles were from four continents across 13 countries, including Egypt, Turkey, Israel, India, China, Taiwan, Japan, Singapore, United States, Canada, Ireland, The Netherlands and Germany. Mean follow-up from selected articles ranged from 15.0 to 174.0 months. Campanacci grading data were available for 809 patients, and grades 1, 2 and 3 were confirmed in 6.4%, 45.8% and 47.8% patients, respectively. Among the site of lesions, most of the lesions were observed in the meta-epiphyseal region of the long bone, mainly at the distal femur (31.6%), followed by proximal tibia (21.5%), radius and ulna (11.36%), proximal femur (5.99%), and sacrum (5.37%) (Table S2).

### 3.3. Quality Assessment

**Figure 2:**
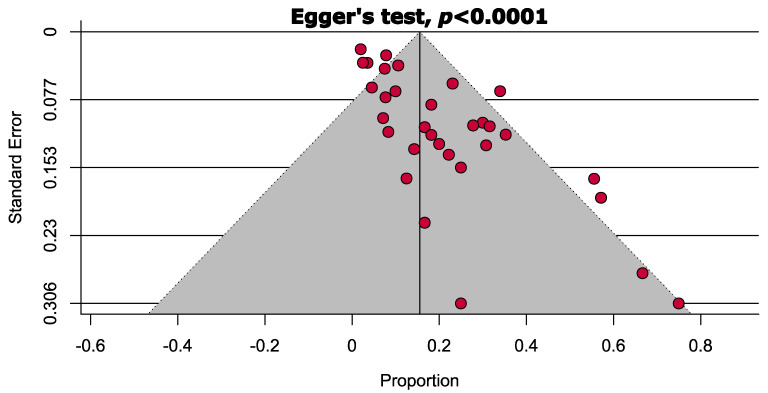
Publication bias assessing the prevalence of local recurrence following cryosurgery of giant cell tumour of bone.

Table S3 contains a comprehensive quality assessment of the included articles. In summary, 7.9% of the articles included were of high quality (low risk of bias), 65.8% were of moderate quality (moderate risk of bias), and 26.3% were of low quality (high risk of bias). Visual examination of the funnel plot and Egger’s test revealed a substantial publication bias (*p* = 0.0001; Figure 2).

### 3.4. Outcomes

**Figure 3:**
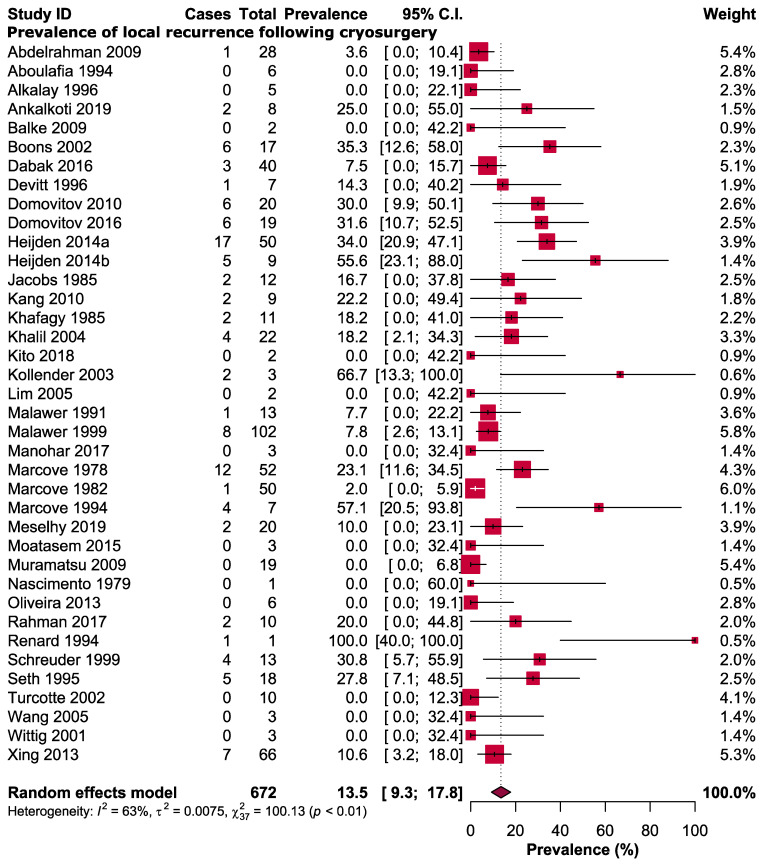
Prevalence of local recurrence following cryosurgery of giant cell tumour of bone.

**Figure 4:**
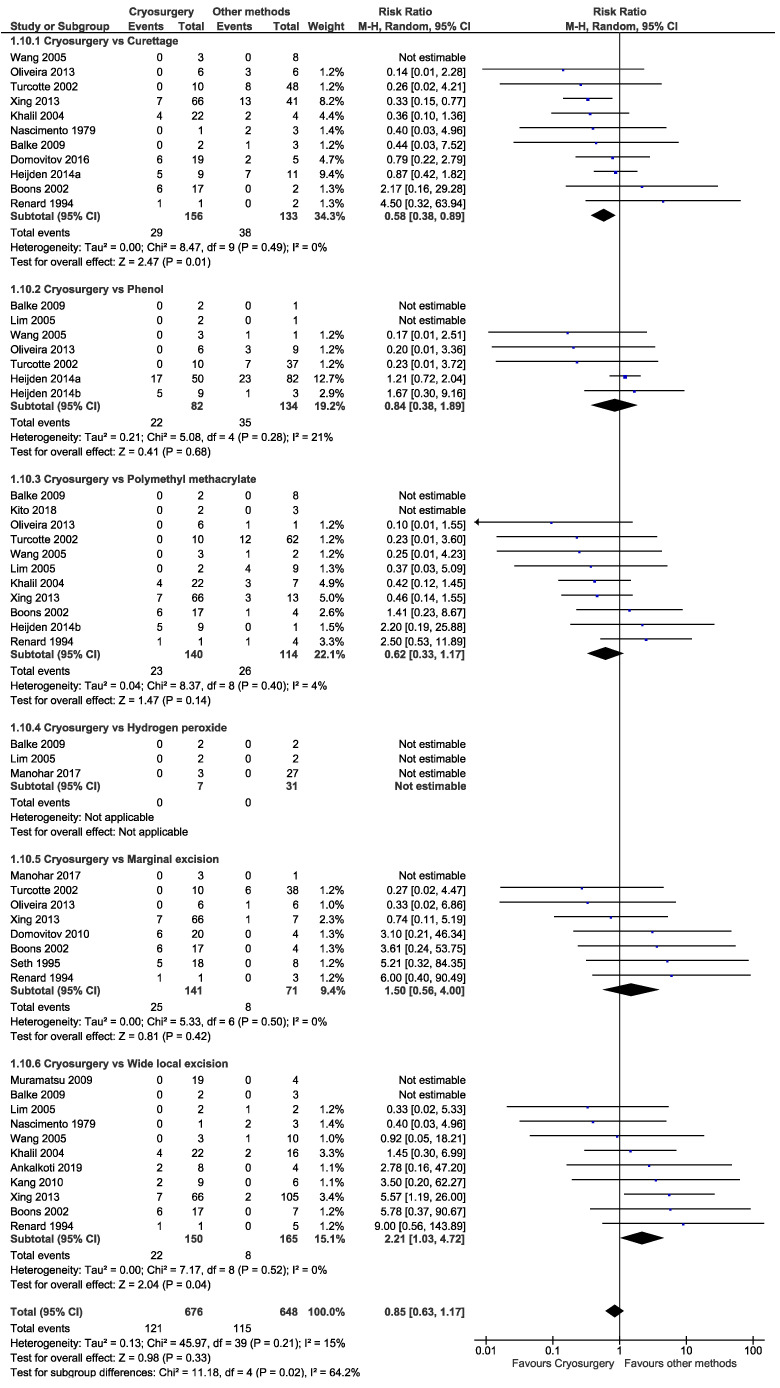
Risk of developing local recurrence followed by cryosurgery vs other methods in giant cell tumours.

The pooled prevalence of local recurrence in giant cell tumours following cryosurgery was 13.5% (95% CI: 9.3–17.8, *I*^2^ = 63%) (Figure 3). The overall risk ratio (RR) of developing local recurrence following cryosurgery was 0.85 (95% CI: 0.63–1.17, *I*^2^ = 15%), which is in favour of cryosurgery; however, statistically not significant (*p* = 0.33) (Figure 4). Three types of local adjuvant were included in the analysis: phenol, PMMA, and hydrogen peroxide. When comparing cryosurgery to phenol and PMMA, the meta-analysis revealed the outcomes in favour of cryosurgery for local control; however statistically not significant, with RR of 0.84 (95% CI: 0.38–1.89, *p* = 0.68) and 0.62 (95% CI: 0.33–1.17, *p* = 0.14), respectively (Figure 4). The hydrogen peroxide group had no local recurrence, and the risk ratio could not be determined since both groups had no local recurrence. The study included two types of extralesional excision: marginal excision and wide local excision. When comparing cryosurgery with marginal excision, the RR favoured the marginal group, where the risk ratio was 1.50 (95% CI: 0.56–4.00); however statistically not significant (*p* = 0.42). Wide local excision groups were found efficient for local control, and we observed the RR as 2.21 (95% CI: 1.03–4.72), which was statistically significant (*p* = 0.04) (Figure 4).

In our study, for the age-based subgroup analysis, we divided the age of the patients into three groups. Group A: 20–30 years old, group B: 31–40 years old and group C: more than 40 years old. Interestingly, we observed that the prevalence of local recurrence Increased with the growing age of the patients. We identified the pooled prevalence of local recurrence of giant cell tumour following cryosurgery in group A as 14.5% (95% CI: 3.8–25.1, *I*^2^ = 63%), group B as 15.4% (95% CI: 9.3–21.4, *I*^2^ = 66%), and group C as 22.5% (95% CI: 0.0–63.4, *I*^2^ = 62%). Based on the location of the participants, European subjects exhibited the highest prevalence of local recurrence of the giant cell tumour following the cryosurgery 24.2% (95% CI: 11.2–37.3, *I*^2^ = 73%) followed by North American 13.4% (95% CI: 6.8–20.0, *I*^2^ = 67%), African 7.9% (95% CI: 2.4–13.4, *I*^2^ = 2%), and Asian 5.1% (95% CI: 0.0–10.3, *I*^2^ = 4%) (Table 2 and Figure S1).

The main complications following cryosurgery in giant cell tumours that we identified were (i) fracture 3.9% (95% CI: 1.5–6.4, *I*^2^ = 21%), (ii) infection 4.0% (95% CI: 1.4–6.6, *I*^2^ = 35%), (iii) nerve injury 2.1% (95% CI: 0.1–4.1, *I*^2^ = 23%) and (iv) skin necrosis 1.5% (95% CI: 0.1–3.0, *I*^2^ = 0%) (Table 2 and Figure S2).

**Figure 5:**
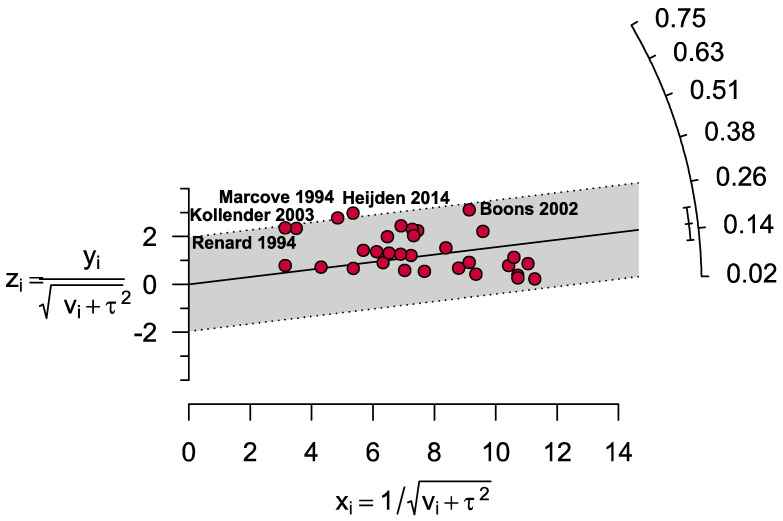
Galbraith’s plot identified five outlier studies.

Based on the Galbraith plot, five studies [26,36,42,48,52] were identified as an outlier and thus possible sources of heterogeneity (Figure 5). Although low to moderate levels of heterogeneity were observed in the primary and subgroup analyses estimating the prevalence (between 2% and 73%), low levels of heterogeneity were detected, estimating the risk of local recurrence in the cryosurgery group versus other treatment types (0–21%). From the sensitivity analyses, we detected that after excluding outlier studies (2.9% lower), small studies (0.2% lower), and low-quality studies (0.2% higher), the result of the main finding did not alter substantially (Figure S3) indicating the reliability and robustness of our estimated prevalence of local recurrence of giant cell tumour of bone following cryosurgery.

## 4. Discussion

Giant cell tumours have been the most difficult to treat among the aggressive benign tumours because of the high local recurrence rate following curettage [39]. The ideal surgical outcome is when the tumour is excised with tumour-free margins, low surgical morbidity and good functional outcomes. Surgical options for giant cell tumours can be either by excision or curettage, with or without local adjuvants, depending on the involvement of the joint surface. The use of different adjuvant therapies is still controversial, and there is no clear consensus for treating giant cell tumours following curettage. Therefore, our primary objective of this systematic review and meta-analysis was to estimate the prevalence of local recurrence of giant cell tumours of bone following liquid nitrogen as adjuvant or cryosurgery.

Liquid nitrogen causes necrosis in tissues by forming intracellular ice crystals and disrupting membranes. Furthermore, repetitive cycles of rapid freezing and slow thawing improve surgical margins by up to 2 cm, similar to marginal resection [43,56]. In our study, the local recurrence rate following cryosurgeries was low, with the pooled prevalence of local recurrence being 13.5% from 38 included articles. A total of 24 articles used the open technique, which was described by Marcove et al. [56], either by direct pour technique or pressurised spray liquid nitrogen in cryosurgery; one article utilised a closed technique described by Hicky and Jacob et al. [31] and 13 articles did not explicitly describe any technique. In addition, bisphosphonates or denosumab were not given in any of the included studies, and only one study [29] underwent radiotherapy before or after the cryosurgeries.

Our study shows no significant difference among the three treatment modalities: phenol, polymethyl methacrylate (PMMA), and hydrogen peroxide. Phenol is used as a chemical adjuvant, causing protein coagulation on the surface of the curetted cavity [26]. The disadvantages of phenol were mainly severe systemic toxicity and carcinogenic potential for the surgeon by inhalation [57]. PMMA is used as a thermal adjuvant, improving the margin up to 0.5 mm in the cortical bone and 1.5–2.0 mm in the cancellous bone [58]. In addition, PMMA is used together with other adjuvants for osseous reconstruction in weight-bearing bones. Heijden et al. observed that the recurrence rates in the phenol group were 19% (3–34%), and if PMMA was used as a sole adjuvant, the recurrence rate was 20% (0–29) [5]. In addition, argon beam coagulation is only available in one article [52], with a small sample size; thus, it was not included in the analysis.

Cryosurgery limitations include large and high-grade malignant bone tumours extending into the soft tissue. Furthermore, applying liquid nitrogen directly to the soft tissues can cause cellular damage to the nearby tissue and neurovascular structures [59]. Gage et al. 1966 introduced the closed techniques where the liquid nitrogen was delivered through the minimal invasive tube to treat malignant soft tissue lesions [60]. However, the disadvantage of this closed technique is the inability to kill tumour cells at the periphery and contamination [61]. Cryogel is a new method associated with good control of local recurrence and less complication; however, there was no reported case using this new method in treating giant cell tumours.

Cryosurgery has several complications. Thus, it is not preferred by many surgeons. Our studies revealed local complications such as fracture, infection, nerve injury, and skin necrosis following cryosurgery of giant cell tumours of bone. Postoperative fracture is the most common complication, and our study showed that the pooled prevalence of fracture following cryosurgery is 3.9% of 474 patients from 26 included articles. Fracture after cryosurgery is commonly due to the significant bone defect in the weight-bearing bones and bone necrosis, causing delayed bone healing [22,62]. In addition, the number of freeze-thaw cycles is also associated with fracture. Most authors recommend two cycles to achieve local control, which carried no significant benefit and caused a higher rate of fracture and non-unions [31,63].

Deep and superficial infection rates are low, with a pool prevalence of 4.0% (1.4–6.6%), with most patients being treated with antibiotics. Liquid nitrogen in touch with the skin causes skin necrosis; however, the risk is minimised when the liquid nitrogen is appropriately handled, and regular irrigation of the surrounding tissues with warm saline is recommended.

Nerve injury is the most complication when cryosurgery is used in the sacrum. Domovitov et al. [29] observed that from 19 patients who had cryosurgery in the sacrum, six patients had pre-existing neurology such as neurogenic bowel and bladder, erectile dysfunction, and weakness; neurology status remained the same after the cryosurgery. However, none of the patients developed new-onset neurology after the cryosurgery; in fact, 12 patients had neurogenic or sciatica pain before the surgery and the symptoms resolved after the cryosurgery. Marcove et al. [42] and Heijden et al., 2014 [52] observed that 14.3–66.6% of patients had nerve palsy following cryosurgery in the sacrum, and 0–50% of them experienced permanent nerve injury. Overall, this meta-analysis’s pooled prevalence of nerve injury was 2.1% from 25 studies.

The advantage of extralesional excision of giant cell tumours involving the joint is that it can eradicate the disease but result in poor functional outcomes. Resection of the pelvic, sacrum, coccyx, distal ulna, proximal radius, and fibula, tubular bones of the hand and foot is indicated when reconstruction is not possible, such as in pathological fractures and large lesions with a cortical breach which is insufficient to retain cement [4]. Our study shows no significant difference in recurrence rate comparing cryosurgery with marginal excision (*p* = 0.42) but significantly different from wide local excision (*p* = 0.04). Thus, we recommend intralesional excision with adjuvant therapy to treat giant cell tumours, and cryosurgeries are options.

The study’s strength is that it is the first meta-analysis to comprehensively analyse the prevalence of local recurrence of giant cell tumours of bone following cryosurgeries. This meta-analysis includes a large number of articles and hence a large number of patients, resulting in a more accurate estimation. Our sensitivity analyses confirmed that the main outcome is reliable and robust. Nonetheless, there are some limitations, such as—although our study had the opportunity to compare with other local adjuvants; however, it may not represent the respective cohort because our search strategies focused on only cryosurgery. Another limitation of our study is that most studies are moderate to low-quality studies, and this is because most of the studies are cohort studies, and the treatment is based on tumour characteristics and the surgeon’s preference. In addition, the functional outcome is not analysed in our study due to the variety in assessing the function of the limb post cryosurgery. Even though overall complications were low, only a small number of patients were reported using cryosurgery in the GCT of the pelvis and sacrum. Thus, treatment for GCT in the pelvis and sacrum has remained a challenge, and most surgeons prefer treatment with denosumab due to its complexity and the risk of nerve injury.

## 5. Conclusions

This meta-analysis found that the local recurrence of giant cell tumours after cryosurgery was 13.5%, with a low complication rate. In our meta-analysis, comparing cryosurgery with other local adjuvants (i.e., phenol, PMMA, and hydrogen peroxide) or marginal excision showed no significant difference in the local recurrence rate. Thus, cryosurgery is one of the treatment options for local control of the recurrence of giant cell tumours while preserving limb function.

## Figures and Tables

**Table 1:** Major characteristics of the included studies.

No	Study ID [References]	Study Duration	Country	Mean Age	Total Number of GCT Patients (Male%)	Mean Follow-Up (Months)	Campanacci Grade		
							1	2	3
1	Abdelrahman 2009 [22]	2005–2008	Egypt	36.3	28 (35.7)	34.0	10	14	4
2	Aboulafia 1994 [23]	1984–1990	United States	NR	6 (NR)	54.3	0	6	0
3	Alkalay 1996 [24]	NR	Israel	27.2	5 (60.0)	31.6	0	5	0
4	Ankalkoti 2019 [4]	2009–2016	India	31.3	12 (58.3)	NR	3	2	7
5	Balke 2009 [25]	1980–2008	Germany	42.4	20 (40.0)	47.9	0	3	15
6	Boons 2002 [26]	NR	The Netherlands	34.0	36 (52.8)	121.3	NR	NR	NR
7	Dabak 2016 [27]	2006–2013	Turkey	33.0	40 (47.5)	43.0	9	25	6
8	Devitt 1996 [28]	1986–1993	Ireland	NR	7 (NR)	60.0	NR	NR	NR
9	Domovitov 2010 [30]	1940–2008	United States	38.0	26 (38.5)	147.0	1	12	13
10	Domovitov 2016 [29]	1973–2012	United States	31.8	24 (54.2)	87.0	2	5	17
11	Heijden 2014a [5]	1990–2010	The Netherlands	33.0	132 (52.3)	93.0	NR	NR	NR
12	Heijden 2014b [52]	1990–2010	The Netherlands	41.0	26 (42.3)	98.0	NR	NR	NR
13	Jacobs 1985 [31]	1971–1981	United States	28.0	12 (66.7)	51.0	NR	NR	NR
14	Kang 2010 [32]	1994–2004	United States	38.0	15 (66.7)	60.0	0	0	15
15	Khafagy 1985 [33]	1978–1982	Egypt	31.7	11 (54.5)	15.0	NR	NR	NR
16	Khalil 2004 [34]	1998–2002	Egypt	32.9	52 (26.9)	24.0	NR	NR	R
17	Kito 2018 [35]	1978–1995	Japan	33.0	5 (80.0)	28.1	1	4	0
18	Kollender 2003 [36]	1991–1999	Israel	22.6	3 (66.7)	92.0	NR	NR	NR
19	Lim 2005 [37]	1993–2001	Singapore	33.0	16 (43.7)	64.4	2	4	10
20	Malawer 1991 [39]	1976–1988	United States	NR	13 (NR)	75.5	NR	NR	NR
21	Malawer 1999 [38]	1983–1993	United States	27.0	102 (52.0)	78.0	15	47	40
22	Manohar 2017 [40]	2003–2007	India	NR	32 (50.0)	24.0	NR	NR	NR
23	Marcove 1978 [43]	1965–1977	United States	30.0	52 (34.6)	43.0	NR	NR	NR
24	Marcove 1982 [41]	NR	United States	NR	50 (NR)	NR	NR	NR	NR
25	Marcove 1994 [42]	1973–1992	United States	20.0	7 (28.6)	121.0	NR	NR	NR
26	Meselhy 2019 [44]	2013–2015	Egypt	31.6	20 (40.0)	28.6	4	10	6
27	Moatasem 2015 [45]	2006–2011	Egypt	NR	3 (NR)	40.0	0	1	2
28	Muramatsu 2009 [46]	1988–2007	Japan	38.0	23 (65.2)	45.0	0	14	9
29	Nascimento 1979 [47]	1949–1977	United States	41.0	8 (37.5)	63.0	NR	NR	NR
30	Oliveira 2013 [6]	1987–2010	The Netherlands	29.6	30 (56.7)	94.8	NR	NR	NR
31	Rahman 2017 [7]	2003–2015	Egypt	34.0	10 (40.0)	57.0	NR	NR	NR
32	Renard 1994 [48]	1962–1989	The Netherlands	31.0	19 (57.9)	174.0	NR	NR	NR
33	Schreuder 1999 [49]	NR	The Netherlands	NR	13 (NR)	34.0	NR	NR	NR
34	Seth 1995 [50]	1958–1988	United States	34.0	26 (46.2)	108.0	2	8	16
35	Turcotte 2002 [51]	1983–1998	Canada	36.0	186 (47.3)	57.0	7	100	76
36	Wang 2005 [53]	1983–2001	Taiwan	37.6	24 (50.0)	90.0	0	0	24
37	Wittig 2001 [54]	1992–1997	United States	23.6	3 (100.0)	54.0	NR	NR	NR
38	Xing 2013 [55]	1988–2008	China	32.3	276 (55.1)	64.2	6	124	131

GCT: giant Cell Tumour, NR: Not reported.

**Table 2:** Subgroup analyses estimating the prevalence of local recurrence in different age groups and locations and the prevalence of adverse events followed by cryosurgery.

Subgroups	Prevalence (95% CI)	Total Number of Patients Analysed	Number of Studies Analysed	Heterogeneity	
				*I^2^*	*p*-Value
Based on mean ages					
Group A (Age 20–30 years)	14.5% (3.8–25.1)	190	8	63%	0.02
Group B (Age 31–40 years)	15.4% (9.3–21.4)	375	20	66%	0.0005
Group C (Age > 40 years)	22.5% (0.0–63.4)	12	3	62%	0.31
Based on the location of the patients					
Europe	24.2% (11.2–37.3)	153	11	73%	0.001
North America	13.4% (6.8–20.0)	322	14	67%	0.0003
Africa	7.9% (2.4–13.4)	94	6	2%	0.42
Asia	5.1% (0.0–10.3)	103	7	4%	0.60
Adverse events					
Fracture	3.9% (1.5–6.4)	474	26	21%	0.46
Infection	4.0% (1.4–6.6)	471	25	35%	0.04
Nerve injury	2.1% (0.1–4.1)	471	25	23%	0.13
Skin necrosis	1.5% (0.1–3.0)	471	25	0%	0.85

CI: confidence interval.

## Data Availability

The data presented in this study are available within the article and Supplementary Materials.

## Supplementary Materials

The following are available online at https://www.mdpi.com/article/10.3390/cancers14143338/s1, Table S1: Search strategies, Table S2: Number and percentage of giant cell tumours based on location, Table S3: Quality assessment of the included studies, Figure S1: Prevalence of local recurrence following cryosurgery of giant cell tumour in different age groups (A–C) and patients from different locations (D–G), Figure S2: Adverse events including (A) fracture, (B) infection, (C) nerve injury and (D) skin necrosis observed followed by cryosurgery of giant cell tumour of bone, Figure S3: Sensitivity analyses by (A) excluding outlier, (B) small and (C) low-quality studies.
