# Supplementary material for: Global Prevalence and Risk of Local Recurrence Following Cryosurgery of Giant Cell Tumour of Bone: A Meta-Analysis
Source: Cancers (Basel). 2022 Jul 8;14(14):3338. doi: 10.3390/cancers14143338 (PMC9318769; doi:10.3390/cancers14143338)
Supplement: Supplementary file 1 [file cancers-14-03338-s001.zip › cancers-1790925-supplementary.pdf]

A

Study ID	Cases	Total	Prevalence	95% C.I.	Weight
**Prevalence of local recurrence (Mean age 20-30 years)**					
Alkalay 1996	0	5	0.0	[0.0; 22.1]	12.1%
Jacobs 1985	2	12	16.7	[0.0; 37.8]	12.7%
Kollender 2003	2	3	66.7	[13.3; 100.0]	3.4%
Malawer 1999	8	102	7.8	[2.6; 13.1]	24.1%
Marcove 1978	12	52	23.1	[11.6; 34.5]	19.7%
Marcove 1994	4	7	57.1	[20.5; 93.8]	6.3%
Oliveira 2013	0	6	0.0	[0.0; 19.1]	14.0%
Wittig 2001	0	3	0.0	[0.0; 32.4]	7.6%
**Random effects model**		**190**	**14.5**	**[3.8; 25.1]**	**100.0%**

Heterogeneity: I^2 = 63%, τ^2 = 0.0115, χ^2_7 = 18.87 (p < 0.01)

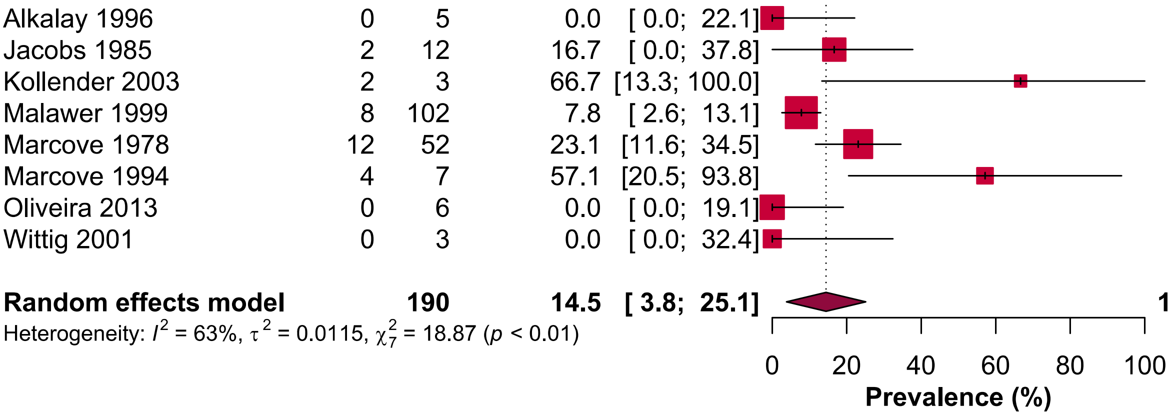

B

Study ID	Cases	Total	Prevalence	95% C.I.	Weight
**Prevalence of local recurrence (Mean age 31-40 years)**					
Abdelrahman 2009	1	28	3.6	[0.0; 10.4]	8.8%
Ankalkoti 2019	2	8	25.0	[0.0; 55.0]	2.9%
Boons 2002	6	17	35.3	[12.6; 58.0]	4.1%
Dabak 2016	3	40	7.5	[0.0; 15.7]	8.4%
Domovitov 2010	6	20	30.0	[9.9; 50.1]	4.7%
Domovitov 2016	6	19	31.6	[10.7; 52.5]	4.5%
Heijden 2014a	17	50	34.0	[20.9; 47.1]	6.8%
Kang 2010	2	9	22.2	[0.0; 49.4]	3.3%
Khafagy 1985	2	11	18.2	[0.0; 41.0]	4.1%
Khalil 2004	4	22	18.2	[2.1; 34.3]	5.8%
Kito 2018	0	2	0.0	[0.0; 42.2]	1.7%
Lim 2005	0	2	0.0	[0.0; 42.2]	1.7%
Meselhy 2019	2	20	10.0	[0.0; 23.1]	6.8%
Muramatsu 2009	0	19	0.0	[0.0; 6.8]	8.8%
Rahman 2017	2	10	20.0	[0.0; 44.8]	3.7%
Renard 1994	1	1	100.0	[40.0; 100.0]	0.9%
Seth 1995	5	18	27.8	[7.1; 48.5]	4.6%
Turcotte 2002	0	10	0.0	[0.0; 12.3]	7.0%
Wang 2005	0	3	0.0	[0.0; 32.4]	2.6%
Xing 2013	7	66	10.6	[3.2; 18.0]	8.6%
**Random effects model**		**375**	**15.4**	**[9.3; 21.4]**	**100.0%**

Heterogeneity: I^2 = 66%, τ^2 = 0.0096, χ^2_{19} = 56.10 (p < 0.01)

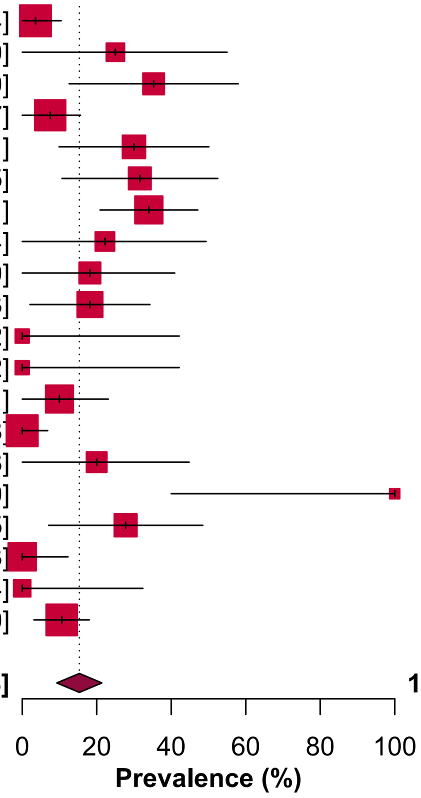

C

Study ID	Cases	Total	Prevalence	95% C.I.	Weight
**Prevalence of local recurrence (Mean age >40 years)**					
Balke 2009	0	2	0.0	[0.0; 42.2]	34.5%
Heijden 2014b	5	9	55.6	[23.1; 88.0]	40.5%
Nascimento 1979	0	1	0.0	[0.0; 60.0]	25.1%
**Random effects model**		**12**	**22.5**	**[0.0; 63.4]**	**100.0%**

Heterogeneity: $I^2 = 62\%$, $\tau^2 = 0.0802$, $\chi^2_2 = 5.28$ ($p = 0.07$)

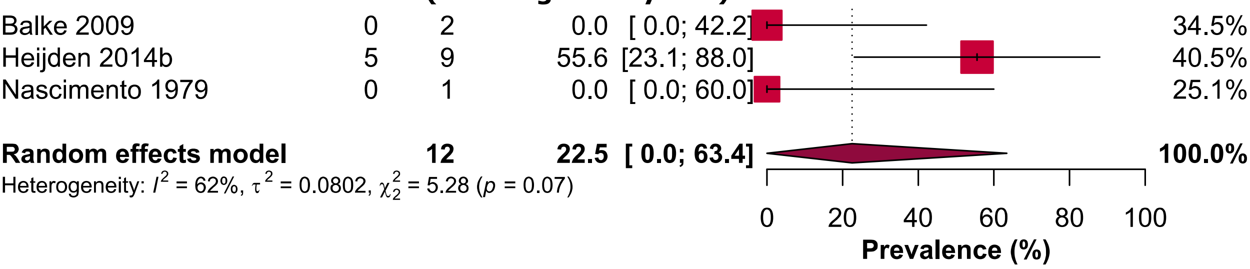

D

Study ID	Cases	Total	Prevalence	95% C.I.	Weight
**Prevalence of local recurrence (Europe)**					
Alkalay 1996	0	5	0.0	[0.0; 22.1]	10.5%
Balke 2009	0	2	0.0	[0.0; 42.2]	5.8%
Boons 2002	6	17	35.3	[12.6; 58.0]	10.3%
Dabak 2016	3	40	7.5	[0.0; 15.7]	14.2%
Devitt 1996	1	7	14.3	[0.0; 40.2]	9.4%
Heijden 2014a	17	50	34.0	[20.9; 47.1]	13.0%
Heijden 2014b	5	9	55.6	[23.1; 88.0]	7.8%
Kollender 2003	2	3	66.7	[13.3; 100.0]	4.3%
Oliveira 2013	0	6	0.0	[0.0; 19.1]	11.4%
Renard 1994	1	1	100.0	[40.0; 100.0]	3.6%
Schreuder 1999	4	13	30.8	[5.7; 55.9]	9.7%
**Random effects model**		**153**	**24.2**	**[11.2; 37.3]**	**100.0%**

Heterogeneity: $I^2 = 73\%$, $\tau^2 = 0.0295$, $\chi^2_{10} = 37.37$ ($p < 0.01$)

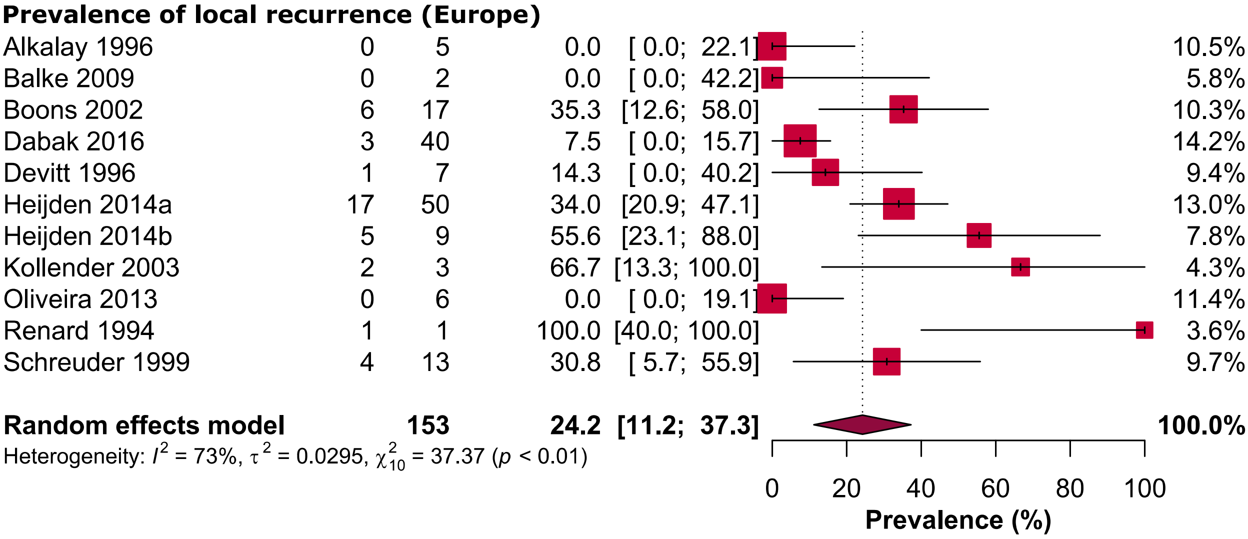

E

Study ID	Cases	Total	Prevalence	95% C.I.	Weight
**Prevalence of local recurrence (North America)**					
Aboulafia 1994	0	6	0.0	[0.0; 19.1]	6.7%
Domovitov 2010	6	20	30.0	[9.9; 50.1]	6.3%
Domovitov 2016	6	19	31.6	[10.7; 52.5]	6.0%
Jacobs 1985	2	12	16.7	[0.0; 37.8]	5.9%
Kang 2010	2	9	22.2	[0.0; 49.4]	4.2%
Malawer 1991	1	13	7.7	[0.0; 22.2]	8.8%
Malawer 1999	8	102	7.8	[2.6; 13.1]	14.0%
Marcove 1978	12	52	23.1	[11.6; 34.5]	10.5%
Marcove 1982	1	50	2.0	[0.0; 5.9]	14.6%
Marcove 1994	4	7	57.1	[20.5; 93.8]	2.6%
Nascimento 1979	0	1	0.0	[0.0; 60.0]	1.1%
Seth 1995	5	18	27.8	[7.1; 48.5]	6.1%
Turcotte 2002	0	10	0.0	[0.0; 12.3]	10.0%
Wittig 2001	0	3	0.0	[0.0; 32.4]	3.2%
**Random effects model**		**322**	**13.4**	**[6.8; 20.0]**	**100.0%**

Heterogeneity: $I^2 = 67\%$, $\tau^2 = 0.0073$, $\chi^2_{13} = 39.47$ ($p < 0.01$)

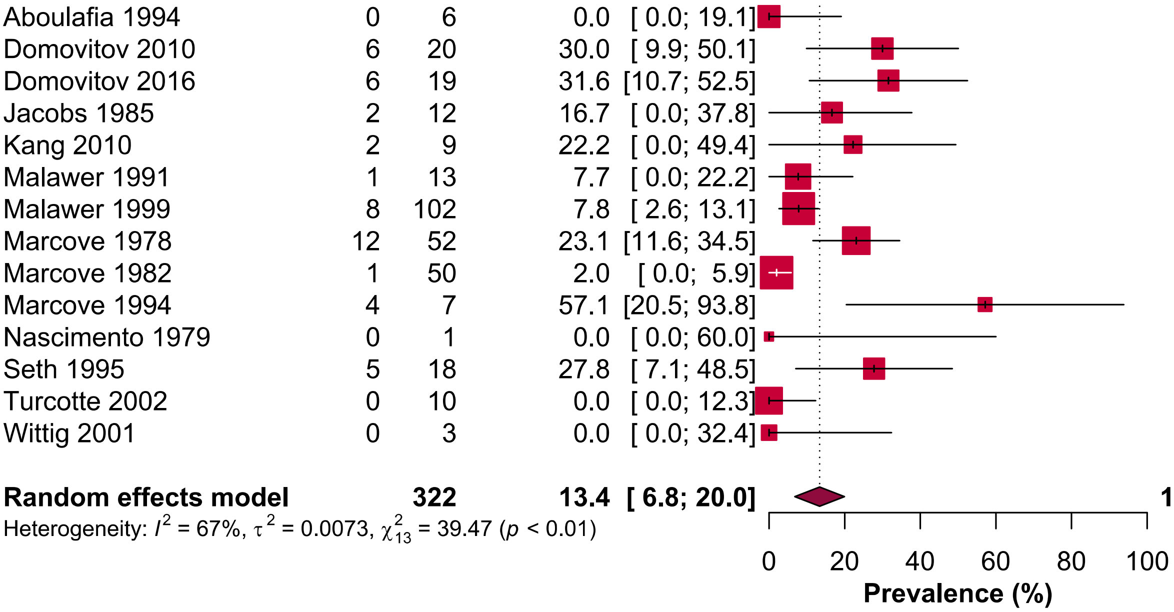

F

Study ID	Cases	Total	Prevalence	95% C.I.	Weight
**Prevalence of local recurrence (Asia)**					
Ankalkoti 2019	2	8	25.0	[0.0; 55.0]	2.9%
Kito 2018	0	2	0.0	[0.0; 42.2]	1.5%
Lim 2005	0	2	0.0	[0.0; 42.2]	1.5%
Manohar 2017	0	3	0.0	[0.0; 32.4]	2.5%
Muramatsu 2009	0	19	0.0	[0.0; 6.8]	47.6%
Wang 2005	0	3	0.0	[0.0; 32.4]	2.5%
Xing 2013	7	66	10.6	[3.2; 18.0]	41.4%
**Random effects model**		**103**	**5.1**	**[0.0; 10.3]**	**100.0%**

Heterogeneity: $I^2 = 4\%$, $\tau^2 = 0.0002$, $\chi^2_6 = 6.24$ ($p = 0.40$)

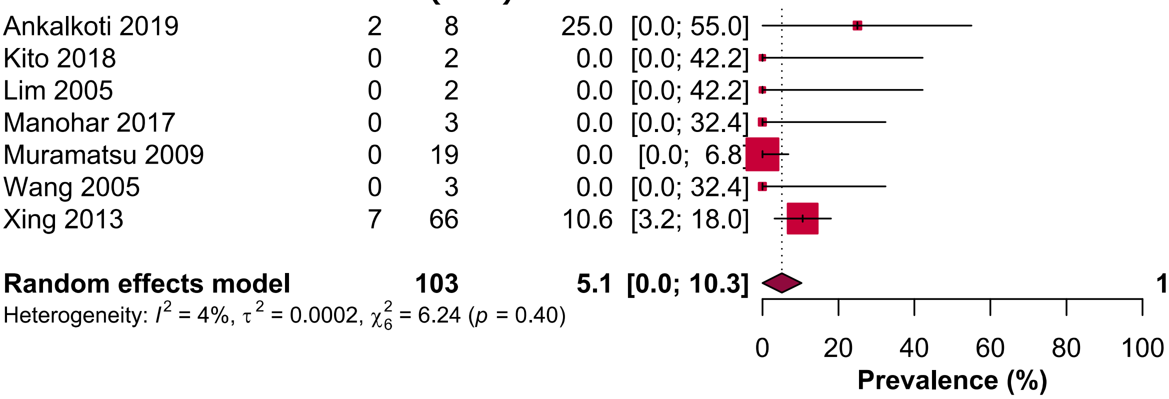

G

Study ID	Cases	Total	Prevalence	95% C.I.	Weight
**Prevalence of local recurrence (Africa)**					
Abdelrahman 2009	1	28	3.6	[0.0; 10.4]	58.0%
Khafagy 1985	2	11	18.2	[0.0; 41.0]	5.8%
Khalil 2004	4	22	18.2	[2.1; 34.3]	11.4%
Meselhy 2019	2	20	10.0	[0.0; 23.1]	17.0%
Moatasem 2015	0	3	0.0	[0.0; 32.4]	2.9%
Rahman 2017	2	10	20.0	[0.0; 44.8]	4.9%
**Random effects model**		**94**	**7.9**	**[2.4; 13.4]**	**100.0%**

Heterogeneity: $I^2 = 2\%$, $\tau^2 = 0.0001$, $\chi^2_5 = 5.10$ ($p = 0.40$)

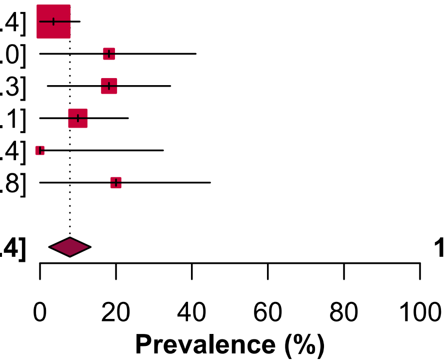

Figure S1. Prevalence of local recurrence following cryosurgery of giant cell tumour in different age groups (A-C) and patients from different locations (D-G).

A

Study ID	Cases	Total	Prevalence	95% C.I.	Weight
**Adverse events (Fracture)**					
Abdelrahman 2009	2	28	7.1	[0.0; 16.7]	5.1%
Aboulafia 1994	0	6	0.0	[0.0; 19.1]	1.5%
Alkalay 1996	0	5	0.0	[0.0; 22.1]	1.2%
Boons 2002	0	17	0.0	[0.0; 7.6]	7.1%
Dabak 2016	1	40	2.5	[0.0; 7.3]	11.8%
Devitt 1996	1	7	14.3	[0.0; 40.2]	0.9%
Domovitov 2016	2	19	10.5	[0.0; 24.3]	2.8%
Heijden 2014a	3	50	6.0	[0.0; 12.6]	8.5%
Heijden 2014b	1	9	11.1	[0.0; 31.6]	1.3%
Jacobs 1985	5	12	41.7	[13.8; 69.6]	0.8%
Kang 2010	0	9	0.0	[0.0; 13.5]	2.9%
Khafagy 1985	1	11	9.1	[0.0; 26.1]	1.9%
Kollender 2003	0	3	0.0	[0.0; 32.4]	0.6%
Lim 2005	0	2	0.0	[0.0; 42.2]	0.3%
Malawer 1999	6	102	5.9	[1.3; 10.4]	12.4%
Marcove 1978	13	52	25.0	[13.2; 36.8]	3.6%
Marcove 1994	0	7	0.0	[0.0; 16.8]	2.0%
Meselhy 2019	0	20	0.0	[0.0; 6.5]	8.6%
Moatasem 2015	0	3	0.0	[0.0; 32.4]	0.6%
Muramatsu 2009	0	19	0.0	[0.0; 6.8]	8.1%
Oliveira 2013	0	6	0.0	[0.0; 19.1]	1.5%
Rahman 2017	0	10	0.0	[0.0; 12.3]	3.4%
Schreuder 1999	0	13	0.0	[0.0; 9.7]	4.9%
Seth 1995	0	18	0.0	[0.0; 7.2]	7.6%
Wang 2005	1	3	33.3	[0.0; 86.7]	0.2%
Wittig 2001	0	3	0.0	[0.0; 32.4]	0.6%
**Random effects model**		**474**	**3.9**	**[1.5; 6.4]**	**100.0%**

Heterogeneity: $I^2 = 21\%$, $\tau^2 = 0.0007$, $\chi^2_{25} = 31.68$ ($p = 0.17$)

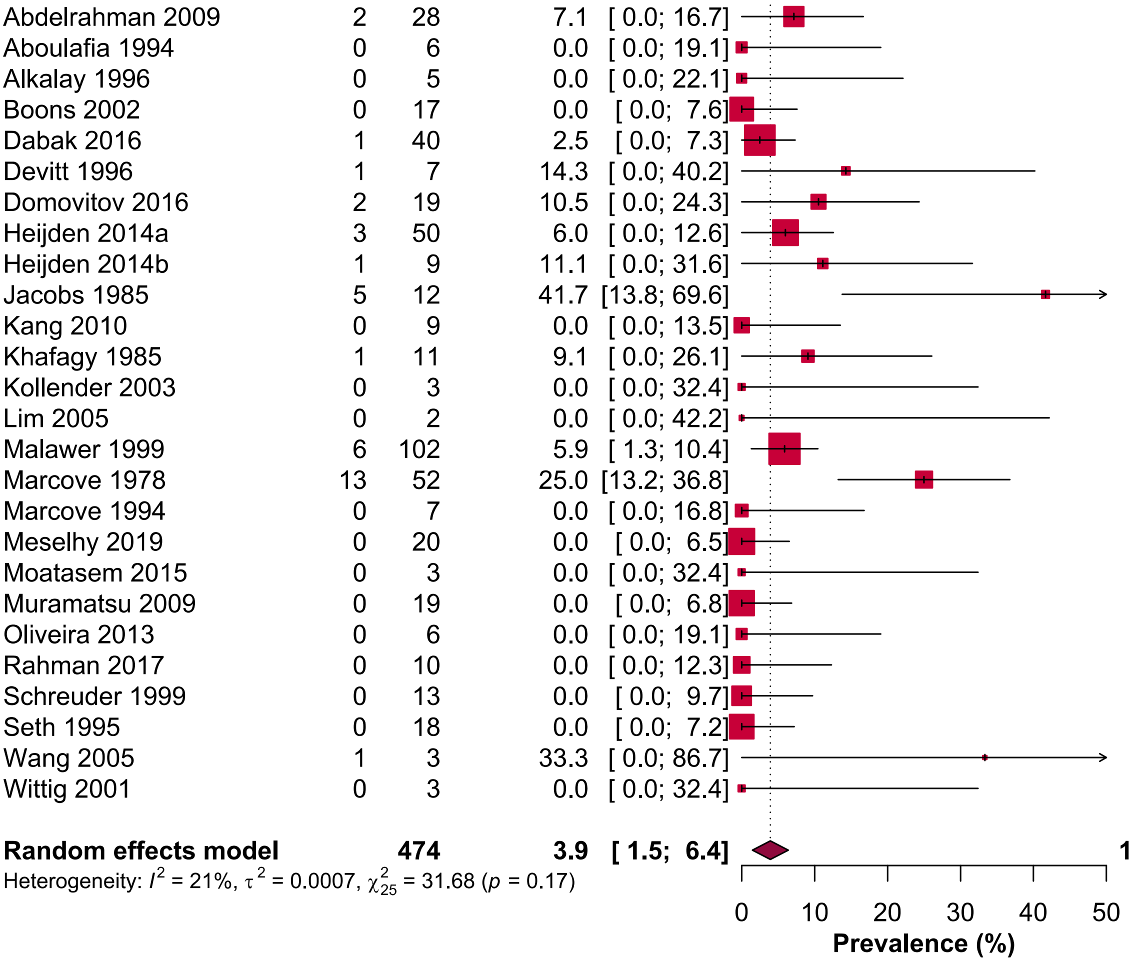

B

Study ID	Cases	Total	Prevalence	95% C.I.	Weight
**Adverse events (Infection)**					
Abdelrahman 2009	1	28	3.6	[0.0; 10.4]	8.2%
Aboulafia 1994	0	6	0.0	[0.0; 19.1]	1.7%
Alkalay 1996	0	5	0.0	[0.0; 22.1]	1.3%
Boons 2002	1	17	5.9	[0.0; 17.1]	4.2%
Dabak 2016	0	40	0.0	[0.0; 3.4]	14.7%
Devitt 1996	2	7	28.6	[0.0; 62.0]	0.6%
Domovitov 2016	6	19	31.6	[10.7; 52.5]	1.4%
Heijden 2014a	3	50	6.0	[0.0; 12.6]	8.6%
Heijden 2014b	1	9	11.1	[0.0; 31.6]	1.5%
Jacobs 1985	2	12	16.7	[0.0; 37.8]	1.4%
Kang 2010	0	9	0.0	[0.0; 13.5]	3.1%
Khafagy 1985	3	11	27.3	[1.0; 53.6]	0.9%
Kollender 2003	0	3	0.0	[0.0; 32.4]	0.6%
Lim 2005	0	2	0.0	[0.0; 42.2]	0.4%
Malawer 1999	0	102	0.0	[0.0; 1.3]	18.6%
Marcove 1978	8	52	15.4	[5.6; 25.2]	5.1%
Marcove 1994	2	7	28.6	[0.0; 62.0]	0.6%
Meselhy 2019	1	20	5.0	[0.0; 14.6]	5.3%
Moatasem 2015	0	3	0.0	[0.0; 32.4]	0.6%
Muramatsu 2009	0	19	0.0	[0.0; 6.8]	8.2%
Oliveira 2013	0	6	0.0	[0.0; 19.1]	1.7%
Rahman 2017	0	10	0.0	[0.0; 12.3]	3.6%
Schreuder 1999	1	13	7.7	[0.0; 22.2]	2.7%
Seth 1995	1	18	5.6	[0.0; 16.1]	4.6%
Wittig 2001	0	3	0.0	[0.0; 32.4]	0.6%
**Random effects model**		**471**	**4.0**	**[1.4; 6.6]**	**100.0%**

Heterogeneity: $I^2 = 35\%$, $\tau^2 = 0.0009$, $\chi^2_{24} = 36.92$ ($p = 0.04$)

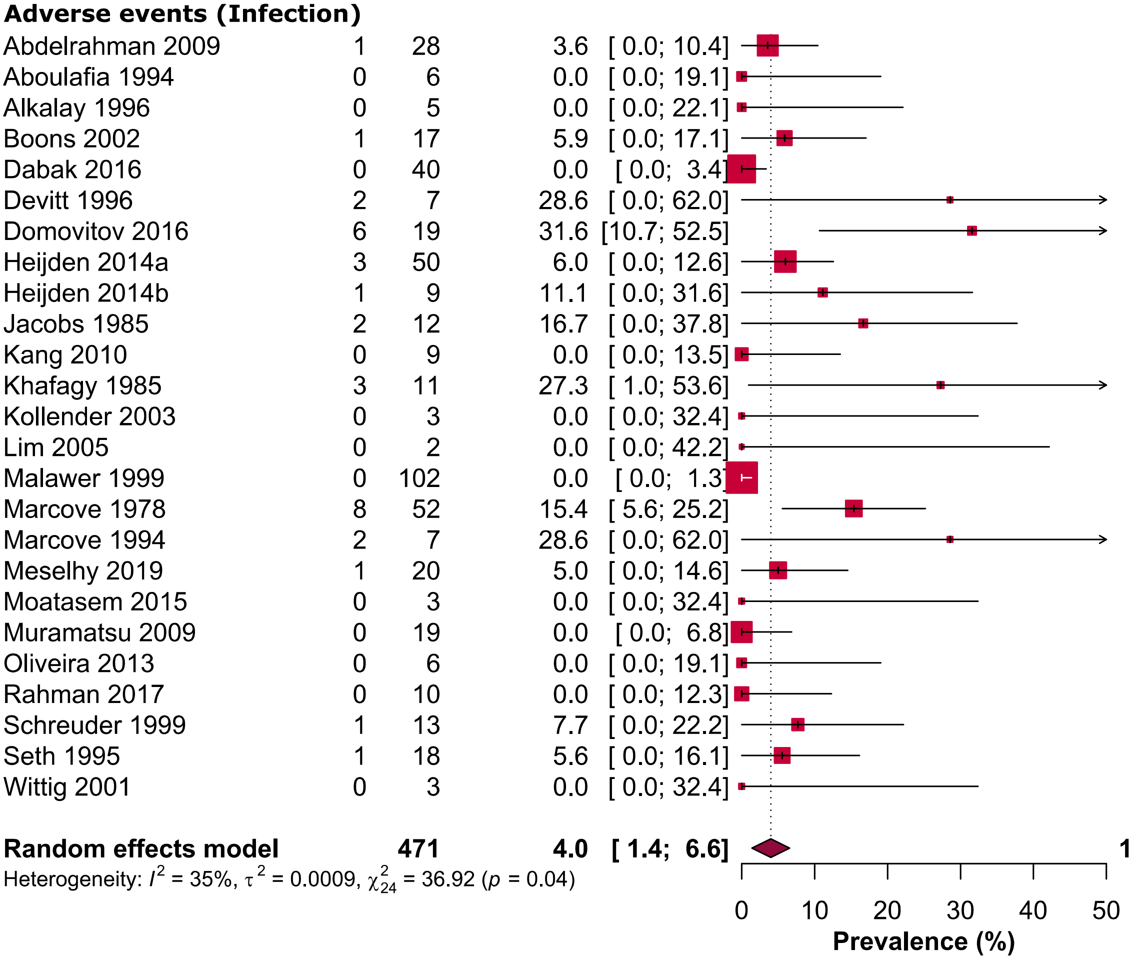

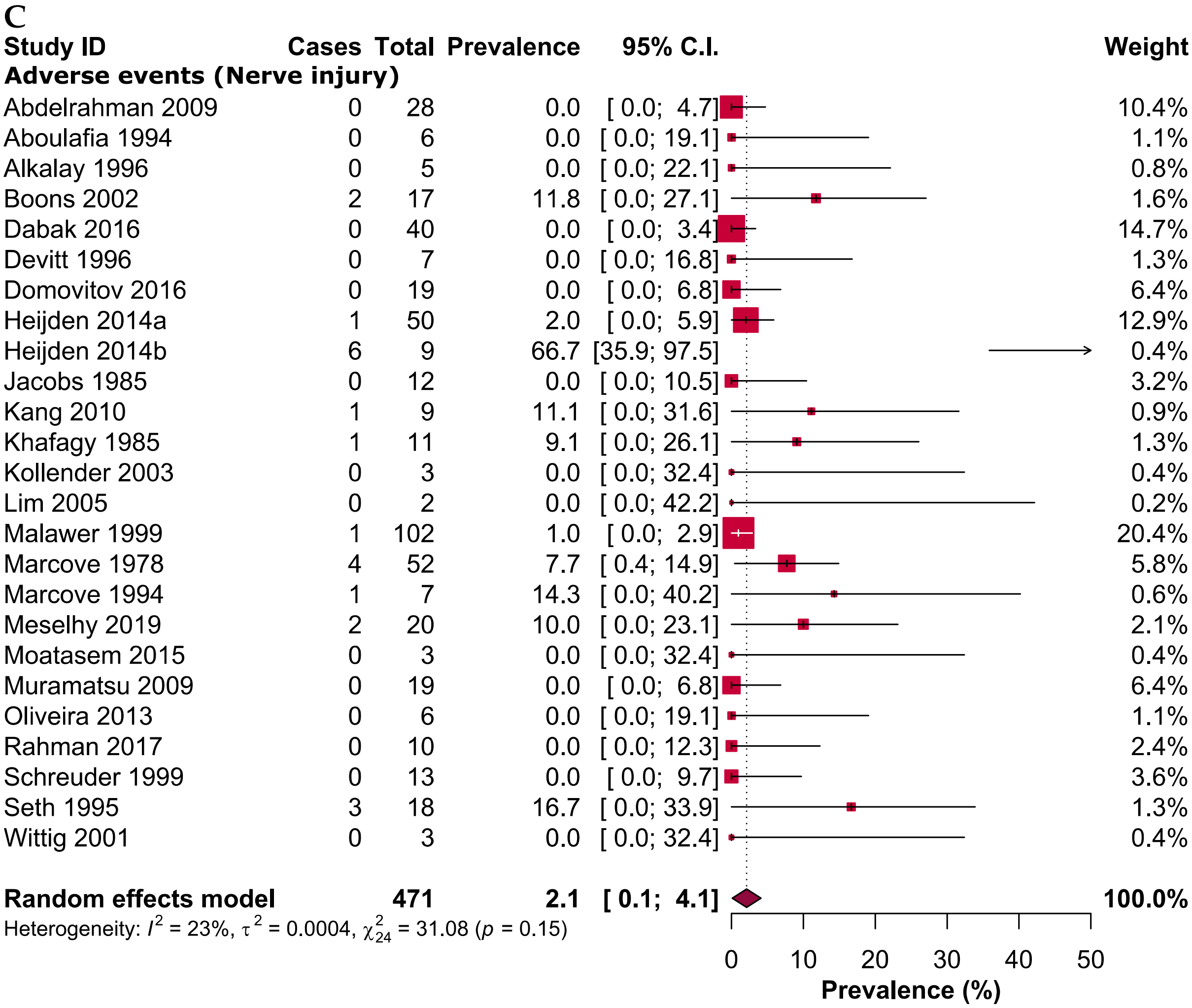
C
Study ID Cases Total Prevalence 95% C.I. Weight
Adverse events (Nerve injury)
Abdelrahman 2009 0 28 0.0 [0.0; 4.7] 10.4%
Aboulafia 1994 0 6 0.0 [0.0; 19.1] 1.1%
Alkalay 1996 0 5 0.0 [0.0; 22.1] 0.8%
Boons 2002 2 17 11.8 [0.0; 27.1] 1.6%
Dabak 2016 0 40 0.0 [0.0; 3.4] 14.7%
Devitt 1996 0 7 0.0 [0.0; 16.8] 1.3%
Domovitov 2016 0 19 0.0 [0.0; 6.8] 6.4%
Heijden 2014a 1 50 2.0 [0.0; 5.9] 12.9%
Heijden 2014b 6 9 66.7 [35.9; 97.5] 0.4%
Jacobs 1985 0 12 0.0 [0.0; 10.5] 3.2%
Kang 2010 1 9 11.1 [0.0; 31.6] 0.9%
Khafagy 1985 1 11 9.1 [0.0; 26.1] 1.3%
Kollender 2003 0 3 0.0 [0.0; 32.4] 0.4%
Lim 2005 0 2 0.0 [0.0; 42.2] 0.2%
Malawer 1999 1 102 1.0 [0.0; 2.9] 20.4%
Marcove 1978 4 52 7.7 [0.4; 14.9] 5.8%
Marcove 1994 1 7 14.3 [0.0; 40.2] 0.6%
Meselhy 2019 2 20 10.0 [0.0; 23.1] 2.1%
Moatasem 2015 0 3 0.0 [0.0; 32.4] 0.4%
Muramatsu 2009 0 19 0.0 [0.0; 6.8] 6.4%
Oliveira 2013 0 6 0.0 [0.0; 19.1] 1.1%
Rahman 2017 0 10 0.0 [0.0; 12.3] 2.4%
Schreuder 1999 0 13 0.0 [0.0; 9.7] 3.6%
Seth 1995 3 18 16.7 [0.0; 33.9] 1.3%
Wittig 2001 0 3 0.0 [0.0; 32.4] 0.4%
Random effects model 471 2.1 [0.1; 4.1] 100.0%
Heterogeneity: I^2 = 23%, τ^2 = 0.0004, χ^2_{24} = 31.08 (p = 0.15)
0 10 20 30 40 50
Prevalence (%)

D

Study ID	Cases	Total	Prevalence	95% C.I.	Weight
**Adverse events (Skin necrosis)**					
Abdelrahman 2009	1	28	3.6	[0.0; 10.4]	4.5%
Aboulafia 1994	0	6	0.0	[0.0; 19.1]	0.6%
Alkalay 1996	0	5	0.0	[0.0; 22.1]	0.4%
Boons 2002	0	17	0.0	[0.0; 7.6]	3.7%
Dabak 2016	0	40	0.0	[0.0; 3.4]	18.8%
Devitt 1996	1	7	14.3	[0.0; 40.2]	0.3%
Domovitov 2016	3	19	15.8	[0.0; 32.2]	0.8%
Heijden 2014a	0	50	0.0	[0.0; 2.7]	29.1%
Heijden 2014b	0	9	0.0	[0.0; 13.5]	1.2%
Jacobs 1985	0	12	0.0	[0.0; 10.5]	1.9%
Kang 2010	0	9	0.0	[0.0; 13.5]	1.2%
Khafagy 1985	0	11	0.0	[0.0; 11.3]	1.7%
Kollender 2003	0	3	0.0	[0.0; 32.4]	0.2%
Lim 2005	0	2	0.0	[0.0; 42.2]	0.1%
Malawer 1999	3	102	2.9	[0.0; 6.2]	19.8%
Marcove 1978	5	52	9.6	[1.6; 17.6]	3.3%
Marcove 1994	2	7	28.6	[0.0; 62.0]	0.2%
Meselhy 2019	0	20	0.0	[0.0; 6.5]	5.0%
Moatasem 2015	0	3	0.0	[0.0; 32.4]	0.2%
Muramatsu 2009	3	19	15.8	[0.0; 32.2]	0.8%
Oliveira 2013	0	6	0.0	[0.0; 19.1]	0.6%
Rahman 2017	0	10	0.0	[0.0; 12.3]	1.4%
Schreuder 1999	0	13	0.0	[0.0; 9.7]	2.3%
Seth 1995	1	18	5.6	[0.0; 16.1]	1.9%
Wittig 2001	1	3	33.3	[0.0; 86.7]	0.1%
**Random effects model**		**471**	**1.5**	**[0.1; 3.0]**	**100.0%**

Heterogeneity: I^2 = 0%, τ^2 = 0, χ^2_{24} = 19.02 (p = 0.75)

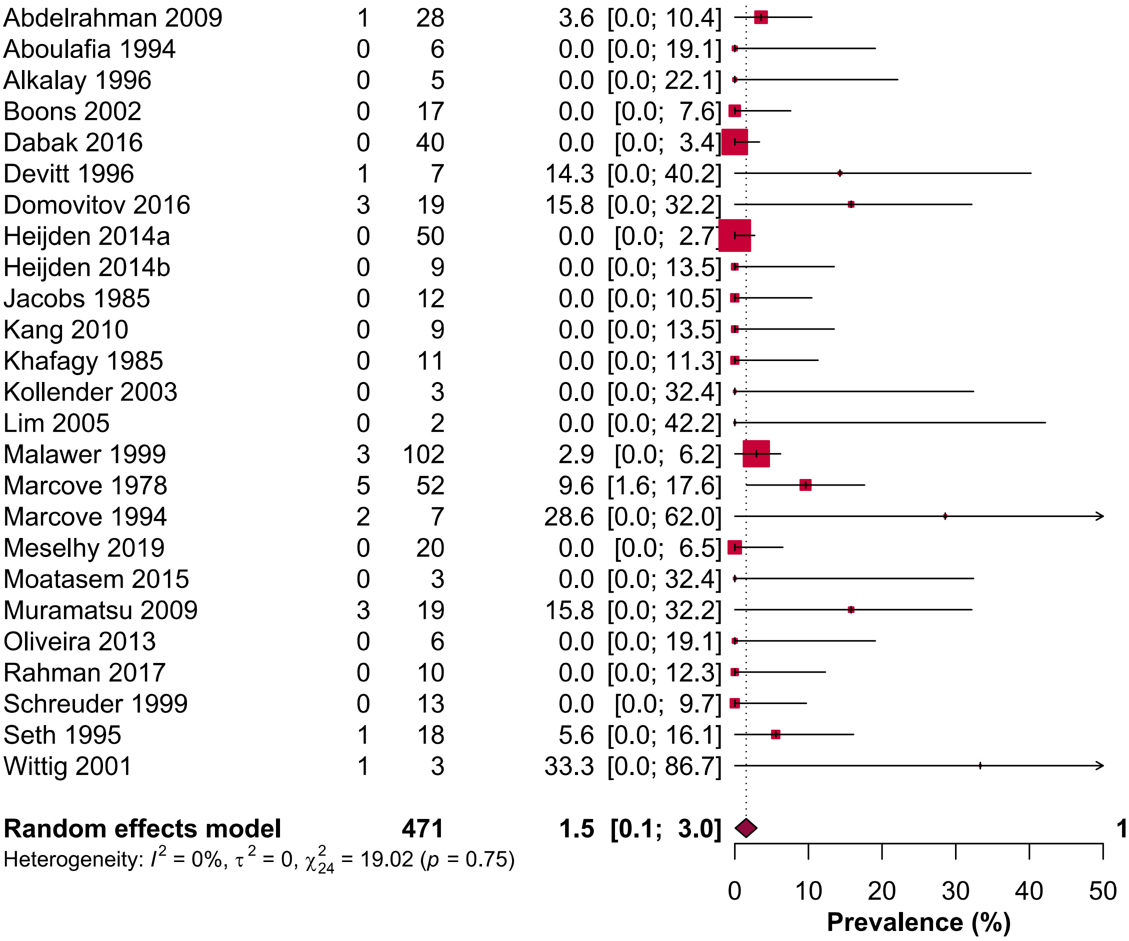

Figure S2. Adverse events including (A) fracture, (B) infection, (C) nerve injury and (D) skin necrosis observed followed by cryosurgery of giant cell tumour of bone.

A

Study ID	Cases	Total	Prevalence	95% C.I.	Weight
**Sensitivity analysis (Excluding outlier studies)**					
Abdelrahman 2009	1	28	3.6	[0.0; 10.4]	6.7%
Aboulafia 1994	0	6	0.0	[0.0; 19.1]	2.6%
Alkalay 1996	0	5	0.0	[0.0; 22.1]	2.1%
Ankalkoti 2019	2	8	25.0	[0.0; 55.0]	1.3%
Balke 2009	0	2	0.0	[0.0; 42.2]	0.7%
Dabak 2016	3	40	7.5	[0.0; 15.7]	6.2%
Devitt 1996	1	7	14.3	[0.0; 40.2]	1.7%
Domovitov 2010	6	20	30.0	[9.9; 50.1]	2.4%
Domovitov 2016	6	19	31.6	[10.7; 52.5]	2.3%
Heijden 2014a	17	50	34.0	[20.9; 47.1]	4.2%
Jacobs 1985	2	12	16.7	[0.0; 37.8]	2.3%
Kang 2010	2	9	22.2	[0.0; 49.4]	1.5%
Khafagy 1985	2	11	18.2	[0.0; 41.0]	2.0%
Khalil 2004	4	22	18.2	[2.1; 34.3]	3.3%
Kito 2018	0	2	0.0	[0.0; 42.2]	0.7%
Lim 2005	0	2	0.0	[0.0; 42.2]	0.7%
Malawer 1991	1	13	7.7	[0.0; 22.2]	3.7%
Malawer 1999	8	102	7.8	[2.6; 13.1]	7.5%
Manohar 2017	0	3	0.0	[0.0; 32.4]	1.1%
Marcove 1978	12	52	23.1	[11.6; 34.5]	4.8%
Marcove 1982	1	50	2.0	[0.0; 5.9]	8.0%
Meselhy 2019	2	20	10.0	[0.0; 23.1]	4.2%
Moatasem 2015	0	3	0.0	[0.0; 32.4]	1.1%
Muramatsu 2009	0	19	0.0	[0.0; 6.8]	6.8%
Nascimento 1979	0	1	0.0	[0.0; 60.0]	0.4%
Oliveira 2013	0	6	0.0	[0.0; 19.1]	2.6%
Rahman 2017	2	10	20.0	[0.0; 44.8]	1.8%
Schreuder 1999	4	13	30.8	[5.7; 55.9]	1.7%
Seth 1995	5	18	27.8	[7.1; 48.5]	2.3%
Turcotte 2002	0	10	0.0	[0.0; 12.3]	4.5%
Wang 2005	0	3	0.0	[0.0; 32.4]	1.1%
Wittig 2001	0	3	0.0	[0.0; 32.4]	1.1%
Xing 2013	7	66	10.6	[3.2; 18.0]	6.5%
**Random effects model**		**635**	**10.6**	**[6.9; 14.3]**	**100.0%**

Heterogeneity: $I^2 = 51\%$, $\tau^2 = 0.0040$, $\chi^2_{32} = 65.27$ ($p < 0.01$)

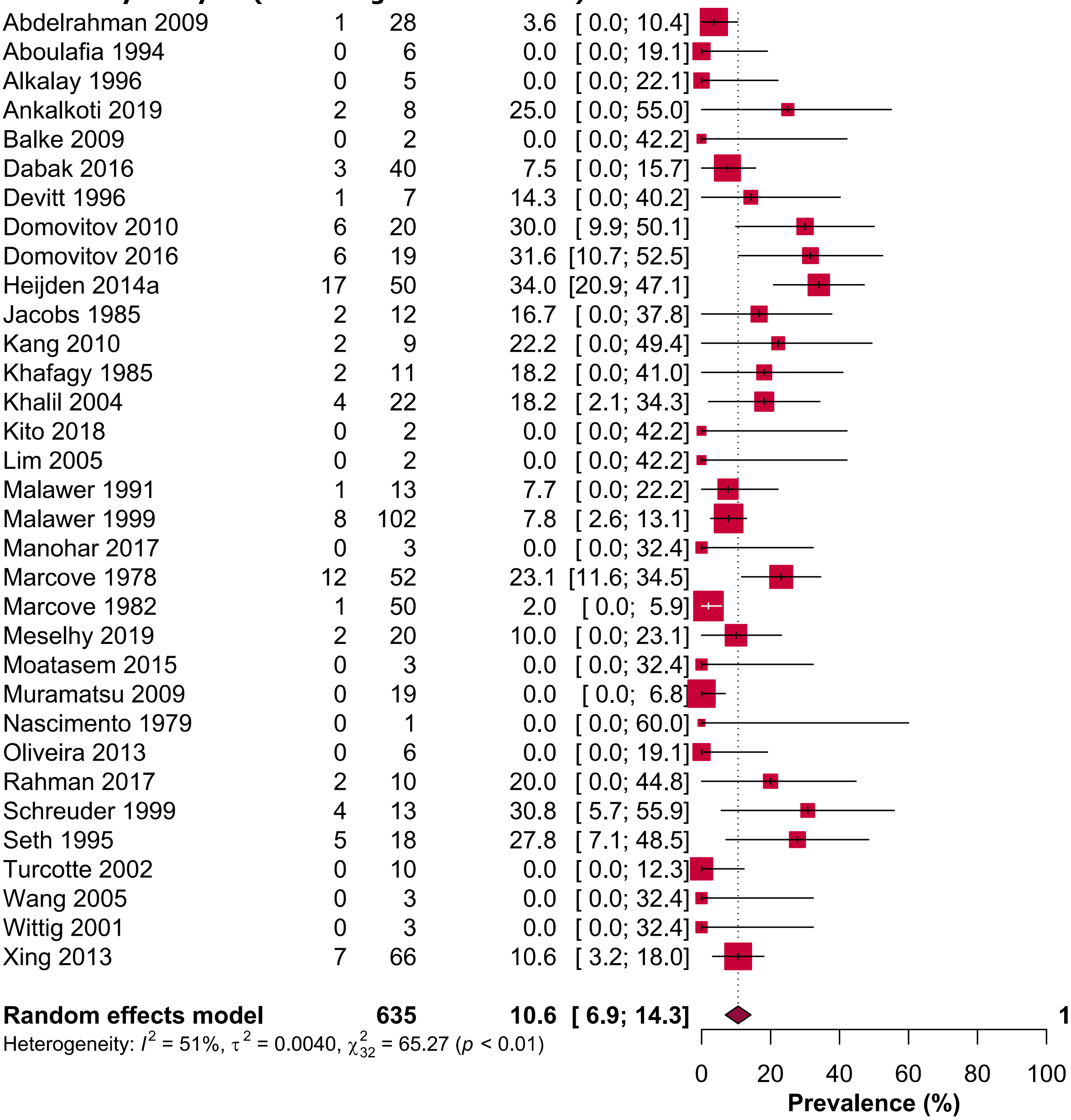

B

Study ID	Cases	Total	Prevalence	95% C.I.	Weight
**Sensitivity analysis (Excluding small studies, n<10)**					
Abdelrahman 2009	1	28	3.6	[0.0; 10.4]	7.5%
Boons 2002	6	17	35.3	[12.6; 58.0]	2.8%
Dabak 2016	3	40	7.5	[0.0; 15.7]	7.0%
Domovitov 2010	6	20	30.0	[9.9; 50.1]	3.3%
Domovitov 2016	6	19	31.6	[10.7; 52.5]	3.2%
Heijden 2014a	17	50	34.0	[20.9; 47.1]	5.2%
Jacobs 1985	2	12	16.7	[0.0; 37.8]	3.1%
Khafagy 1985	2	11	18.2	[0.0; 41.0]	2.8%
Khalil 2004	4	22	18.2	[2.1; 34.3]	4.3%
Malawer 1991	1	13	7.7	[0.0; 22.2]	4.8%
Malawer 1999	8	102	7.8	[2.6; 13.1]	8.1%
Marcove 1978	12	52	23.1	[11.6; 34.5]	5.8%
Marcove 1982	1	50	2.0	[0.0; 5.9]	8.5%
Meselhy 2019	2	20	10.0	[0.0; 23.1]	5.2%
Muramatsu 2009	0	19	0.0	[0.0; 6.8]	7.5%
Rahman 2017	2	10	20.0	[0.0; 44.8]	2.5%
Schreuder 1999	4	13	30.8	[5.7; 55.9]	2.5%
Seth 1995	5	18	27.8	[7.1; 48.5]	3.2%
Turcotte 2002	0	10	0.0	[0.0; 12.3]	5.5%
Xing 2013	7	66	10.6	[3.2; 18.0]	7.3%
**Random effects model**		**592**	**13.3**	**[8.7; 17.9]**	**100.0%**

Heterogeneity: $I^2 = 71\%$, $\tau^2 = 0.0061$, $\chi^2_{19} = 65.45$ ($p < 0.01$)

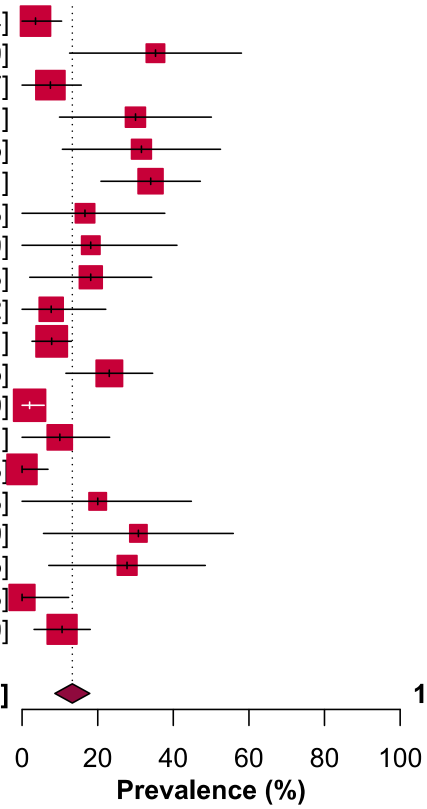

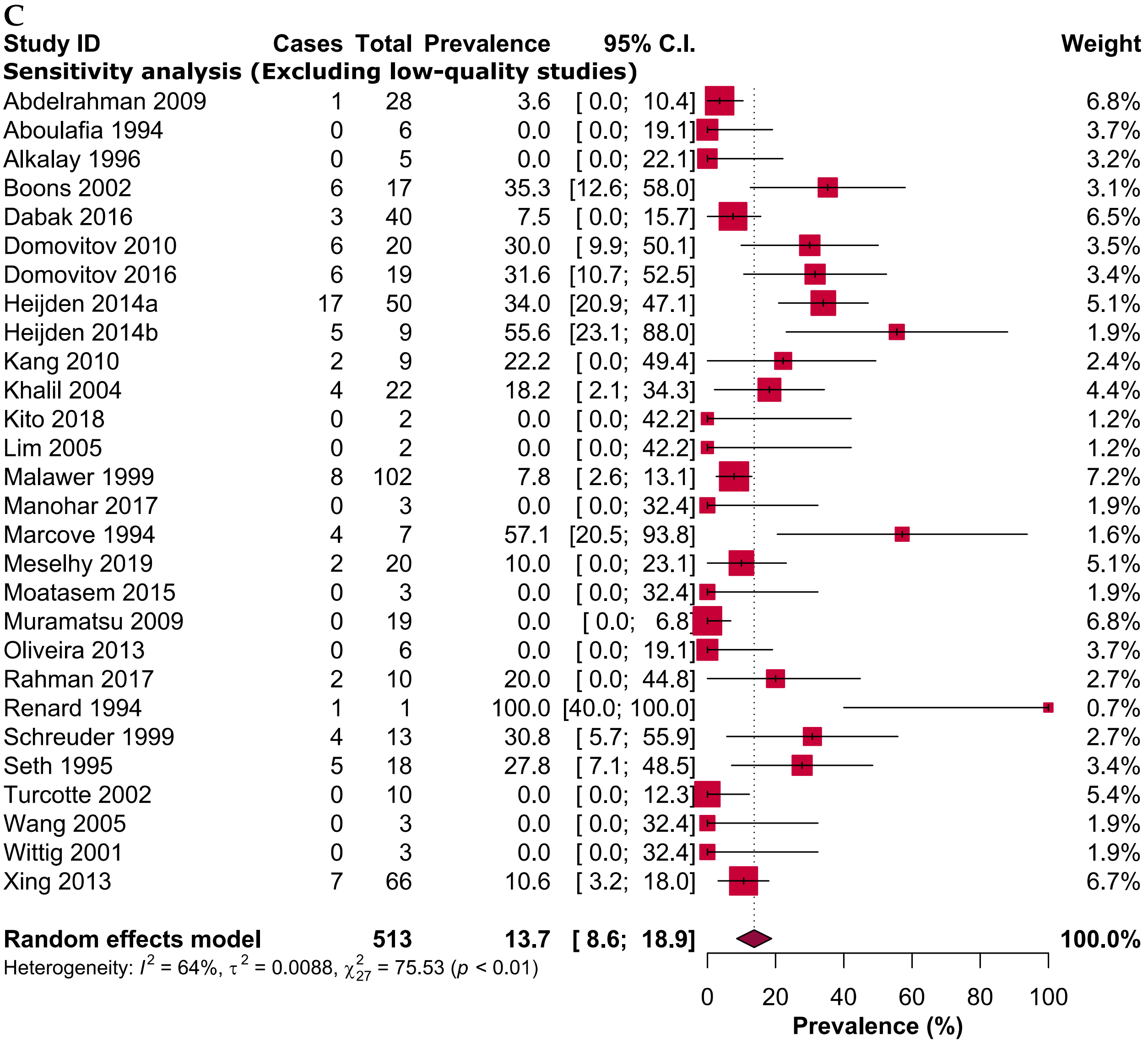

Figure S3. Sensitivity analyses by (A) excluding outlier, (B) small and (C) low-quality studies.

Table S1. Search strategies

Databases	Search strategies
PubMed	(Cryosurgery[Title/Abstract] OR Cryosurgeries[Title/Abstract] OR Cryoablation[Title/Abstract] OR Cryoablations[Title/Abstract] OR Cryotherapy[Title/Abstract] OR Cryotherapies[Title/Abstract] OR Adjuvant[Title/Abstract] OR Liquid nitrogen[Title/Abstract]) AND (Giant cell tumor[Title/Abstract] OR Giant cell tumors[Title/Abstract] OR Giant cell tumour[Title/Abstract] OR Giant cell tumours[Title/Abstract])
Scopus	TITLE-ABS-KEY(Cryosurgery OR Cryosurgeries OR Cryoablation OR Cryoablations OR Cryotherapy OR Cryotherapies OR Adjuvant OR "Liquid nitrogen") AND TITLE("Giant cell tumor" OR "Giant cell tumors" OR "Giant cell tumour" OR "Giant cell tumours")
Web of Science	TI, AB=(Cryosurgery OR Cryosurgeries OR Cryoablation OR Cryoablations OR Cryotherapy OR Cryotherapies OR Adjuvant OR Liquid nitrogen) AND TI, AB=(Giant cell tumor OR Giant cell tumors OR Giant cell tumour OR Giant cell tumours)<br>Indexes=SCI-EXPANDED, SSCI, A&HCI, CPCI-S, CPCI-SSH, BKCI-S, BKCI-SSH, ESCI Timespan=All years
Google Scholar	allintitle: (Cryosurgery OR Cryosurgeries OR Cryoablation OR Cryoablations OR Cryotherapy OR Cryotherapies OR Adjuvant OR “Liquid nitrogen”) (“Giant cell tumor” OR “Giant cell tumors” OR “Giant cell tumour” OR “Giant cell tumours”)
ScienceDirect	Title, abstract, keywords: (Cryosurgery OR Cryosurgeries OR Cryoablation OR Cryoablations OR Cryotherapy OR Cryotherapies OR Adjuvant OR "Liquid nitrogen") AND ("Giant cell tumor" OR "Giant cell tumors" OR "Giant cell tumour" OR "Giant cell tumours")

Table S2. Number and percentage of giant cell tumours based on location

No	Site	Total	Percent
1	Proximal Femur	78	5.99%
2	Distal Femur	412	31.62%
3	Proximal Tibia	281	21.57%
4	Distal Tibia	64	4.91%
5	Fibula	51	3.91%
6	Sacral	70	5.37%
7	Pelvic	36	2.76%
8	Radius/Ulna	148	11.36%
9	Humerus	67	5.14%
10	Hand	40	3.07%
11	Scapula/Clavicle	4	0.31%
12	Foot	43	3.30%
13	Others	9	0.69%

Table S3. Quality assessment of the included studies

No.	Study ID	JBI Critical appraisal checklist for cohort studies											Yes (%)
		1	2	3	4	5	6	7	8	9	10	11	
1	Abdelrahman 2009	NA	NA	Y	N	N	NA	Y	Y	Y	NA	N	57.1
2	Aboulafia 1994	NA	NA	Y	N	N	NA	Y	Y	Y	NA	N	57.1
3	Alkalay 1996	NA	NA	Y	N	N	NA	Y	Y	Y	NA	N	57.1
4	Ankalkoti 2019	Y	N	Y	N	N	NA	Y	U	U	U	Y	40.0
5	Balke 2009	Y	N	Y	N	N	NA	U	Y	Y	NA	N	44.4
6	Boons 2002	Y	N	Y	N	N	NA	Y	Y	Y	NA	Y	66.7
7	Dabak 2016	NA	NA	Y	N	N	NA	Y	Y	Y	NA	Y	71.4
8	Devitt 1996	NA	NA	Y	N	N	NA	U	Y	Y	NA	N	42.9
9	Domovitov 2010	Y	Y	Y	Y	Y	NA	Y	Y	Y	NA	Y	100.0
10	Domovitov 2016	Y	N	Y	N	N	NA	Y	Y	Y	NA	Y	66.7
11	Heijden 2014a	Y	Y	Y	Y	Y	NA	Y	Y	U	U	Y	80.0
12	Heijden 2014b	Y	N	Y	N	N	NA	Y	Y	Y	U	Y	60.0
13	Jacobs 1985	NA	NA	Y	N	N	NA	Y	Y	U	U	N	37.5
14	Kang 2010	Y	Y	Y	N	N	NA	Y	Y	Y	U	Y	70.0
15	Khafagy 1985	NA	NA	Y	N	N	NA	Y	Y	U	U	N	37.5
16	Khalil 2004	Y	N	Y	N	N	NA	Y	Y	Y	NA	N	55.6
17	Kito 2018	Y	N	Y	N	N	NA	Y	Y	Y	NA	N	55.6
18	Kollender 2003	NA	NA	Y	N	N	NA	U	Y	U	U	N	25.0
19	Lim 2005	Y	N	Y	N	N	NA	Y	Y	Y	NA	N	55.6
20	Malawer 1991	NA	NA	Y	N	N	NA	U	Y	Y	NA	N	42.9
21	Malawer 1999	NA	NA	Y	N	N	NA	Y	Y	Y	NA	N	57.1
22	Manohar 2017	Y	N	Y	N	N	NA	Y	Y	Y	NA	N	55.6
23	Marcove 1978	NA	NA	Y	N	N	NA	Y	Y	U	U	N	37.5

24	Marcove 1982	NA	NA	Y	N	N	NA	Y	Y	U	U	N	37.5
25	Marcove 1994	NA	NA	Y	N	N	NA	Y	Y	Y	NA	N	57.1
26	Meselhy 2019	NA	NA	Y	N	N	NA	Y	Y	Y	NA	N	57.1
27	Moatasem 2015	NA	NA	Y	N	N	NA	Y	Y	Y	NA	N	57.1
28	Muramatsu 2009	Y	N	Y	N	N	NA	Y	Y	Y	NA	N	55.6
29	Nascimento 1979	Y	N	Y	N	N	NA	U	Y	Y	NA	N	44.4
30	Oliveira 2013	Y	N	Y	N	N	NA	Y	Y	Y	NA	Y	66.7
31	Rahman 2017	NA	NA	Y	N	N	NA	Y	Y	Y	NA	N	57.1
32	Renard 1994	Y	N	Y	N	N	NA	Y	Y	Y	NA	N	55.6
33	Schreuder 1999	NA	NA	Y	N	N	NA	Y	Y	Y	NA	N	57.1
34	Seth 1995	Y	N	Y	N	N	NA	Y	Y	Y	Y	N	60.0
35	Turcotte 2002	Y	N	Y	N	N	NA	Y	Y	Y	NA	Y	66.7
36	Wang 2005	Y	N	Y	N	N	NA	Y	Y	U	N	Y	50.0
37	Wittig 2001	NA	NA	Y	N	N	NA	Y	Y	Y	Y	N	62.5
38	Xing 2013	Y	N	Y	N	N	NA	Y	Y	Y	NA	Y	66.7

1. Were the two groups similar and recruited from the same population? 2. Were the exposures measured similarly to assign people to both exposed and unexposed groups? 3. Was the exposure measured in a valid and reliable way? 4. Were confounding factors identified? 5. Were strategies to deal with confounding factors stated? 6. Were the groups/participants free of the outcome at the start of the study (or at the moment of exposure)? 7. Were the outcomes measured in a valid and reliable way? 8. Was the follow up time reported and sufficient to be long enough for outcomes to occur? 9. Was follow up complete, and if not, were the reasons to loss to follow up described and explored? 10. Were strategies to address incomplete follow up utilized? 11. Was appropriate statistical analysis used? Y=Yes; N=No; U=Unclear; NA: Not applicable.
